# Global Epidemiology of Ischemic Heart Disease: Results from the Global Burden of Disease Study

**DOI:** 10.7759/cureus.9349

**Published:** 2020-07-23

**Authors:** Moien AB Khan, Muhammad Jawad Hashim, Halla Mustafa, May Yousif Baniyas, Shaikha Khalid Buti Mohamad Al Suwaidi, Rana AlKatheeri, Fatmah Mohamed Khalfan Alblooshi, Meera Eisa Ali Hassan Almatrooshi, Mariam Eisa Hazeem Alzaabi, Reem Saif Al Darmaki, Shamsa Nasser Ali Hussain Lootah

**Affiliations:** 1 Family Medicine, College of Medicine and Health Sciences, United Arab Emirates University, Al Ain, ARE; 2 Primary Care, North West London - National Health Service Provider, London, GBR; 3 Family Medicine, College of Medicine and Health Sciences, United Arab Emirates University, Al Ain, GBR

**Keywords:** ischemic heart disease, coronary artery disease, atherosclerotic cardiovascular disease, epidemiology, burden of disease, cardiovascular diseases, non-communicable diseases, global burden

## Abstract

Background

Ischemic heart disease (IHD) is a leading cause of death worldwide. Also referred to as coronary artery disease (CAD) and atherosclerotic cardiovascular disease (ACD), it manifests clinically as myocardial infarction and ischemic cardiomyopathy. This study aims to evaluate the epidemiological trends of IHD globally.

Methods

The most up-to-date epidemiological data from the Global Burden of Disease (GBD) dataset were analyzed. GBD collates data from a large number of sources, including research studies, hospital registries, and government reports. This dataset includes annual figures from 1990 to 2017 for IHD in all countries and regions. We analyzed the incidence, prevalence, and disability-adjusted life years (DALY) for IHD. Forecasting for the next two decades was conducted using the Statistical Package for the Social Sciences (SPSS) Time Series Modeler (IBM Corp., Armonk, NY).

Results

Our study estimated that globally, IHD affects around 126 million individuals (1,655 per 100,000), which is approximately 1.72% of the world’s population. Nine million deaths were caused by IHD globally. Men were more commonly affected than women, and incidence typically started in the fourth decade and increased with age. The global prevalence of IHD is rising. We estimated that the current prevalence rate of 1,655 per 100,000 population is expected to exceed 1,845 by the year 2030. Eastern European countries are sustaining the highest prevalence. Age-standardized rates, which remove the effect of population changes over time, have decreased in many regions.

Conclusions

IHD is the number one cause of death, disability, and human suffering globally. Age-adjusted rates show a promising decrease. However, health systems have to manage an increasing number of cases due to population aging.

## Introduction

Cardiovascular diseases cause approximately one-third of deaths worldwide [1]. Among cardiovascular illnesses, ischemic heart disease (IHD) ranks as the most prevalent [2]. Indeed, IHD is acknowledged as an important threat to sustainable development in the 21st century [3]. Also referred to as coronary artery disease (CAD) and atherosclerotic cardiovascular disease (ACD), IHD manifests clinically as myocardial infarction and ischemic cardiomyopathy. An increasing number of individuals with non-fatal IHD live with chronic disabilities and impaired quality of life [4]. The primary pathological process that leads to IHD is atherosclerosis, an inflammatory disease of the arteries associated with lipid deposition and metabolic alterations due to multiple risk factors. More than 70% of at-risk individuals have multiple risk factors for IHD, and only 2%-7% of the general population have no risk factors [5].

The increasing incidence of IHD is expected to continue, due not only to the increased prevalence of obesity, diabetes, and metabolic syndrome but also to population aging [6]. The past two decades have witnessed a steep rise in global population aging [7]. Indeed, the United Nations estimates an increase in the population aged over 65 years from one in 11 in 2019 to one in six by 2050 [8]. Emerging issues with social relationships, psychological distress, and less than six hours of sleep a night also contribute to IHD in the current generation [9-10]. Rapid urbanization and globalization in the lower and middle-income countries (LMIC) have led to a shift in disease-related deaths and disabilities from infectious disease to non-communicable diseases such as IHD [4-5].

The financial impact of IHD stems from hospitalizations, treatments, revascularization procedures, clinic visits, emergency visits, and prescribed drug treatments [11]. According to the World Heart Federation, the global cost of CVD in 2010 was approximately US$863 billion, which is expected to rise to more than US$1 trillion by 2030. In countries like the United States, the cost of IHD is approaching 1%-1.5% of the gross domestic product (GDP), with costs per episode of IHD of more than $5,000 (7,13). Notably, the median total cost of IHD care in low and middle-income countries (LMIC) country-specific health expenditure per capita was 10% of the total healthcare expenditure [12].

Despite the high prevalence, morbidity, and mortality of IHD, relatively few studies have quantified current epidemiological trends and global forecasts for IHD. For health policy-makers to develop effective and timely strategies to address IHD, an up-to-date analysis is needed. This study aims to analyze the global epidemiology of IHD based on the most recent data and to estimate its future trends.

## Materials and methods

Current epidemiological data on IHD from the Global Burden of Disease (GBD) dataset were analyzed in this study [13]. The GBD dataset has several attractive features: it is actively maintained and updated based on research data and published epidemiological studies and governmental publications from more than 90,000 sources. According to empirical data, it builds models and statistical estimates for health loss due to illness, injury, and risk factors. Annually, the GBD produces prevalence, incidence, death, and disability-adjusted life years (DALYs) measures, which are used to estimate the overall burden of IHD. DALYs are calculated by adding YLLs (years of life lost due to premature death) and YLDs (years of life lost due to disability). It is considered a more rigorous measure of the human impact of disease than simple prevalence or mortality rates.

For this study, the latest data from GBD, the 2017 update, was used. This includes annual figures from 1990 to 2017 for IHD in almost all countries and regions. No regions or countries were excluded. Based on geographical divisions, GBD divides the world into four regions: Asia, Europe, America, and Africa. We analyzed data for all countries in these four regions of the GBD dataset. Age-adjusted rates were used to compare annual rates from 1990 to 2017, to compensate for the effects of changes in population structure and aging. The Guidelines for Accurate and Transparent Health Estimates Reporting (GATHER) guidelines were used to guide transparent data analysis and reporting (Table 1) [14]. We analyzed selected countries within the GBD's four world regions based upon the prevalence rates and geographical size.

**Table 1:** Checklist of information while reporting global health estimates of Ischemic Heart Disease This checklist is used in conjunction with the GATHER statement and Explanation and Elaboration document, found on gather-statement.org. GATHER: Guidelines for Accurate and Transparent Health Estimates Reporting

Item #	Checklist item: Checklist of information that should be included in new reports of global health estimates	Reported in section
Objectives and funding		
1	Define the indicator(s), populations (including age, sex, and geographic entities), and time period(s) for which estimates were made.	Methods
2	List the funding sources for the work.	Acknowledgment
Data Inputs		
For all data inputs from multiple sources that are synthesized as part of the study:		
3	Describe how the data were identified and how the data were accessed.	Methods
4	Specify the inclusion and exclusion criteria. Identify all ad-hoc exclusions.	Methods
5	Provide information on all included data sources and their main characteristics. For each data source used, report reference information or contact name/institution, the population represented, data collection method, year(s) of data collection, sex and age range, diagnostic criteria or measurement method, and sample size, as relevant.	Methods
6	Identify and describe any categories of input data that have potentially important biases (e.g., based on characteristics listed in item 5).	Discussions - Limitations
For data inputs that contribute to the analysis but were not synthesized as part of the study:		
7	Describe and give sources for any other data inputs.	N/A
For all data inputs:		
8	Provide all data inputs in a file format from which data can be efficiently extracted (e.g., a spreadsheet rather than a PDF), including all relevant meta-data listed in item 5. For any data inputs that cannot be shared because of ethical or legal reasons, such as third-party ownership, provide a contact name or the name of the institution that retains the right to the data.	Open Data
Data analysis		
9	Provide a conceptual overview of the data analysis method. A diagram may be helpful.	Methods
10	Provide a detailed description of all steps of the analysis, including mathematical formulae. This description should cover, as relevant, data cleaning, data pre-processing, data adjustments and weighting of data sources, and mathematical or statistical model(s).	Methods
11	Describe how candidate models were evaluated and how the final model(s) were selected.	N/A
12	Provide the results of an evaluation of model performance, if done, as well as the results of any relevant sensitivity analysis.	N/A
13	Describe methods for calculating the uncertainty of the estimates. State which sources of uncertainty were, and were not, accounted for in the uncertainty analysis.	N/A
14	State how analytic or statistical source code used to generate estimates can be accessed.	N/A
Results and Discussion		
15	Provide published estimates in a file format from which data can be efficiently extracted.	Table 1
16	Report a quantitative measure of the uncertainty of the estimates (e.g. uncertainty intervals).	Web reference
17	Interpret results in light of existing evidence. If updating a previous set of estimates, describe the reasons for changes in estimates.	Discussions - Limitations
18	Discuss the limitations of the estimates. Include a discussion of any modeling assumptions or data limitations that affect the interpretation of the estimates.	Discussions - Limitations

Statistical data analysis

The Statistical Package for the Social Sciences (IBM Corp., Armonk, NY) was used for statistical analysis. The Time Series Modeler was used to develop a forecast model using the Expert Modeler option without any events. The stationary R-square was used to measure the goodness of fit. None of the observed values was marked as outliers. There were no missing values in this dataset.

## Results

In 2017, we observed that IHD affects around 126 million individuals globally (1,655 per 100,000), which is estimated to be 1.72% of the world’s population (Table 2).

**Table 2:** Selected countries from each global region. All figures are rates per 100,000 population for 2017. The rates have not been standardized for differences in underlying population age distributions All figures are rates per 100,000 population for 2017. Global Burden of Disease (GBD) four world regions with selected countries based on the prevalence rates and geographical size.

Region	Prevalence (rate per 100,000)	Disability - Adjusted Life Years (rate per 100,000)
Global	1,655	2,228
Europe	3,547	3,771
Germany	3,432	2,855
France	2,696	1,237
Italy	3,468	1,831
Spain	2,733	1,503
Netherlands	3,502	1,451
Switzerland	2,581	1,461
Sweden	3,858	2,192
Turkey	2,418	1,960
Russia	4,198	6,568
United Kingdom	3,337	1,864
Asia and Australasia	1,440	2,272
China	1,612	2,131
India	1,197	2,679
Japan	2,928	1,427
South Korea	1,352	704
Taiwan	1,759	1,241
Saudi Arabia	1,509	1,643
Iran	1,599	2,149
Australia	2,576	1,450
Americas	1,990	1,887
United States	2,929	2,470
Canada	2,335	1,837
Brazil	1,685	1,736
Africa	880	1,309
South Africa	1,227	1,184

In 2017, around nine million deaths were attributed to IHD, making it the leading cause of mortality worldwide. IHD has retained this leading position for more than two decades.

**Figure 1:**
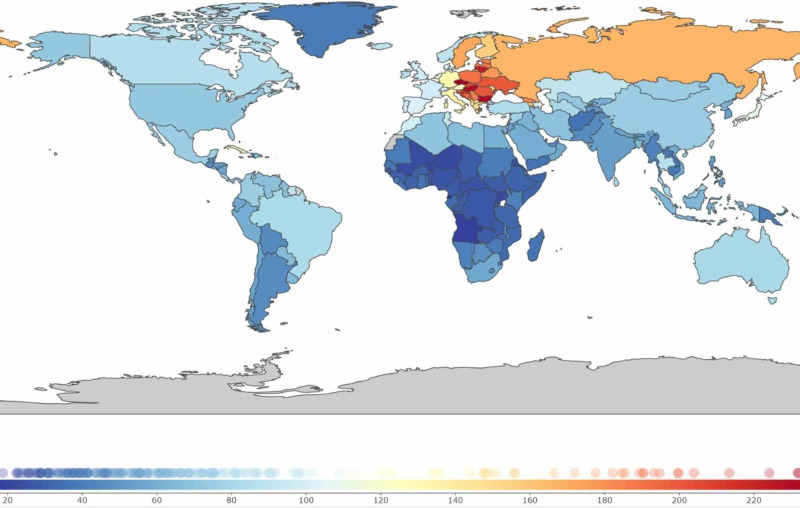
Global distribution of ischemic heart disease Colors indicate prevalence rates per 100,000 population in 2017

**Figure 2:**
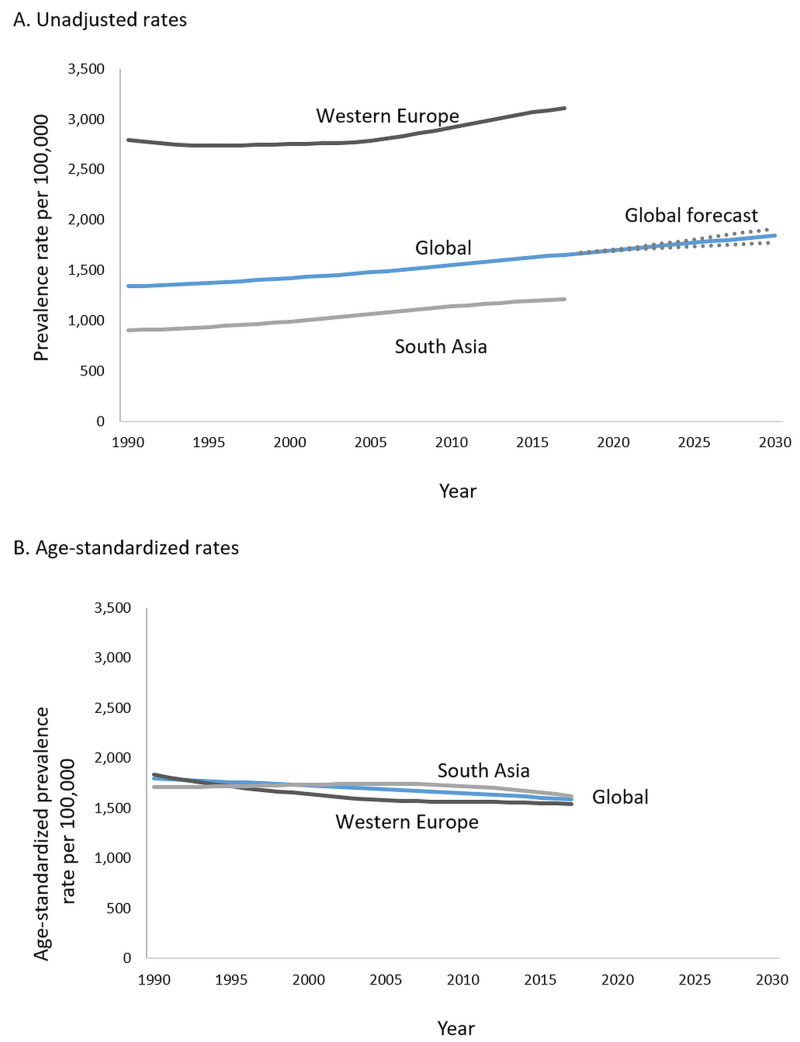
Trends in the prevalence of ischemic heart disease using unadjusted (A) and age-standardized (B) rates Forecast estimates using Statistical Package for the Social Sciences (SPSS) Time-Series Modeler (Ljung Box Q, p = 0.14). Dotted lines indicate upper and lower confidence limits. Age-standardization removes the effect of changes in the population age distribution (such as aging) over time and across global regions. SPSS: IBM Corp. Armonk, NY

In terms of regional distribution, IHD shows the highest prevalence in central and Eastern Europe (Figure 1),

During the last two decades, several Eastern European countries, such as Lithuania, Bulgaria, Latvia, Estonia, and the Czech Republic, have moved up the ranks of highest prevalence. In contrast, high-income countries, such as the United Kingdom, Finland, Denmark, Germany, and Italy, moved down the ranks. Western Europe continues to show an increasing prevalence, substantially higher than South Asia (the Indian subcontinent) and the rest of the world (Figure 2).

Forecasts of prevalence based on predictive models indicate that by 2030, the prevalence of IHD could increase to more than 1,845 per 1000,000, with an upper confidence estimate of 1,917 per 100,000.

**Figure 3:**
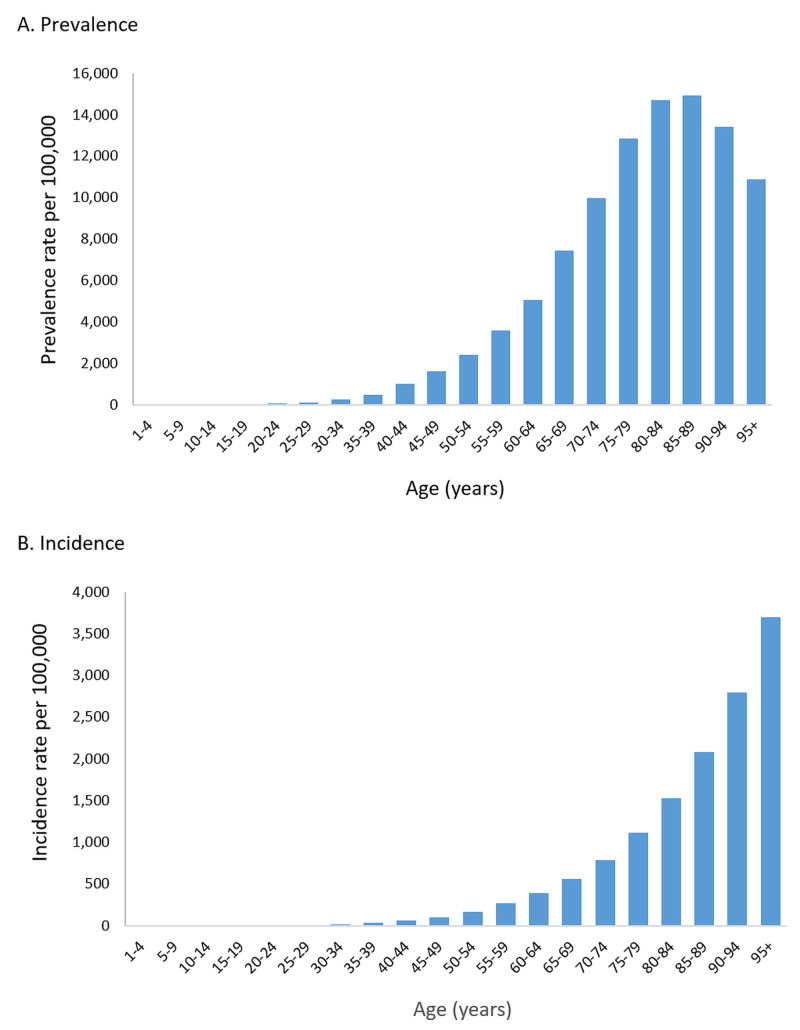
Age distribution of ischemic heart disease worldwide based on prevalence (A) and incidence (B), 2017

Male gender is a well-known risk factor and, consequently, the prevalence of IHD was higher in men as compared to women (1,786 vs. 1,522 cases per 100,000). This difference is present in all age groups. The age of onset also appears to be earlier in men. The age distribution showed expected patterns of the rising prevalence and incidence with increasing age (Figure 3).

Incidence increases from the fourth decade of life and does not decrease after that, reflecting the role of age as a risk factor. Prevalence remains higher than the incidence for all age groups, indicating the chronic nature of IHD.

In terms of human suffering, DALY is a more accurate measure to quantify the burden of disease than prevalence and mortality. In terms of DALY, IHD is the leading cause of disability and years of life lost globally, moving from the fourth position in 1990 to the first position in 2017.

## Discussion

IHD is the leading cause of premature mortality and suffering worldwide, retaining this leading position for decades, albeit with considerable variation across regions. While IHD continues to rise in terms of absolute numbers, age-adjusted rates do show evidence of a decrease.

The number of patients diagnosed with IHD increased over the past 27 years. This increase in CVD burden has serious implications for health systems’ capacity and planning. Particularly, a policy focused on providing tertiary cardiac care may be unsustainable without a corresponding emphasis on prevention and primary care [15]. As this rise is partly due to population increase and aging (the incidence and prevalence of IHD increase with age), age-standardized rates are more indicative of the underlying trends [16]. According to an earlier analysis of GBD data from 1990 to 2010, DALYs increased by 32.4% globally because of population aging and increased by 22.1% because of the increased population [4].

A key finding of our study is that age-standardized rates of incidence, prevalence, mortality, and burden of suffering (as indicated by DALYs) have declined over the past two decades in many regions. This may be due to increasing awareness of lifestyle factors such as cigarette smoking, obesity, and exercise [17]. Public health initiatives have also helped to reduce smoking [18]. National strategies for health promotion can help curtail the obesity epidemic [19]. Although the age-adjusted rates trend down, IHD is by far the leading cause of death [20]. Countries in Eastern Europe show a disproportionately high burden of IHD [17]. This high burden is difficult to attribute to any single factor, however, lifestyle, social stress, and alcoholism have been implicated [17,21-22]. In certain countries, the increased current burden could be attributed to changing population distributions [21,23].

Recent studies relating to the CVD burden have indicated a potential association of increased CVD burden with lower socioeconomic status and less education [24]. Hence, the burden of IHD may be addressed by effective investment in educating the public and creating an awareness of CVD-associated risk factors and early symptoms. Some researchers have suggested that it may be useful to reduce socioeconomic disparity to reduce disease burden [25]. Both age-standardized incidence and mortality have decreased globally in the past 27 years, with high-income countries experiencing the most significant decline. However, the total number of cases will continue to rise due to worsening metabolic risk factors [16]. The forecast of an increased disability burden due to IHD should prompt healthcare policymakers to promote strategies such as putting more emphasis on primary care and primary prevention of IHD. There is a growing concern that the CVD burden is bound to rise due in LMIC to risk factors such as smoking and an unhealthy diet [26-27]. Major challenges of IHD on the health system could be addressed by effective measures to reduce tobacco smoking [28]. Consistent advice on a healthy diet during childhood could also reduce the development of metabolic syndrome in early adulthood [29]. Public health measures, such as reducing total salt intake by one third, could reduce deaths due to IHD by 8% [30].

The main limitation of our study is the reliance on secondary data. The GBD dataset relies on modeling and estimation based on primary data. Real-world data sources can be inaccurate, patchy, and subject to change due to changes in disease classification. Thus, the inferences derived should be considered carefully in light of evolving research evidence. Additionally, GBD does not subdivide IHD into its constituent elements such as myocardial infarction, angina, or heart failure due to ischaemic cardiomyopathy. While it was not possible to analyze acute versus chronic ischemic presentations, the overall burden of illness due to IHD can still be ascertained reasonably accurately. Despite these limitations, this study provides new insights based on the most up-to-date data available, which have not been published elsewhere.

## Conclusions

IHD remains the leading cause of death and premature mortality worldwide, with economic development and urbanization having the greatest impact on disease development. The present analysis provides evidence of a promising decline in age-adjusted rates in many regions. The causes of IHD are well-established, including risk factors such as calorie-dense processed foods, stress, a sedentary lifestyle, and cigarette smoking, so aggressive preventive measures are warranted to control this scourge of modern life.
